# Primary Radial Nerve Lesions in Humerus Shaft Fractures—Revision or Wait and See

**DOI:** 10.3390/jcm13071893

**Published:** 2024-03-25

**Authors:** Alexander Böhringer, Raffael Cintean, Konrad Schütze, Florian Gebhard

**Affiliations:** Department of Trauma Hand and Reconstructive Surgery, Ulm University, Albert-Einstein-Allee 23, 89081 Ulm, Germany

**Keywords:** humeral shaft fractures, primary radial nerve palsy, early nerve exploration, peripheral nerve lesions, nerve recovery, wait-and-see strategy

## Abstract

**Background**: This study investigates the surgical state-of-the-art procedure for humeral shaft fractures with primary radial nerve palsy based on its own case series in relation to the current and established literature. **Methods**: Retrospective review of treated cases between January 2018 and December 2022 describing radial nerve palsy after humerus shaft fractures, radiological fracture classification, intraoperative findings, surgical procedure, patient follow-up and functional outcome. **Results**: A total of 804 patients (463 women and 341 men) with humerus shaft fractures were identified. A total of 33 patients showed symptomatic lesions of the radial nerve (4.1%). The primary lesion was identified in 17 patients (2.1%). A broad and inhomogeneous distribution of fractures according to the AO classification was found. According to the operative reports, the distraction of the radial nerve was found eleven times, bony interposed three times and soft tissue constricted/compressed three times. In every case the radial nerve was surgically explored, there was no case of complete traumatic nerve transection. Four intramedullary nails and thirteen locking plates were used for osteosynthesis. Complete recovery of nerve function was seen in 12 cases within 1 to 36 months. Three patients still showed mild hypesthesia in the thumb area after 18 months. Two patients were lost during follow-up. **Conclusions**: With this study, we support the strategy of early nerve exploration and plate osteosynthesis in humeral fractures with primary radial nerve palsy when there is a clear indication for surgical fracture stabilisation. In addition, early exploration appears sensible in the case of palsies in open fractures and secondary palsy following surgery without nerve exposure as well as in the case of diagnostically recognisable nerve damage. Late nerve exploration is recommended if there are no definite signs of recovery after 6 months. An initial wait-and-see strategy with clinical observation seems reasonable for primary radial nerve palsies without indication for surgical fracture stabilisation.

## 1. Introduction

With an incidence of 82.1/10^5^ and accounting for 7.4% of all adult fractures, humeral fractures are very common. They are caused by both high-energy and low-energy trauma and are then often associated with osteoporosis due to older age and female gender [1]. Lesions of the radial nerve often occur in fractures of the shaft and distal third [2,3]. Several anatomical peculiarities in these areas have been described as a reason for this. The radial nerve and its accompanying vessels run in the middle of the shaft over a length of 6.2 cm with direct bone contact dorsally on the humerus. It then continues distally through the lateral intermuscular septum. Throughout this course, the nerve is poorly mobile until it crosses the ulnar flexure ventrally of the lateral humeral epicondyle protected by muscle [4,5,6]. With a reported incidence of 11.8%, these are the most common peripheral nerve lesions associated with bone fractures [2,3,7], whereby the origin of the damage and the appearance of the clinical symptoms can be primary or secondary. Different morphologic stages of the nerve lesion (neuropraxia, axonotmesis and neurotmesis) can cause different degrees of functional impairment in terms of peripheral numbness and paralysis [8,9]. According to the literature, the overall recovery rate is 88.1%, with 70.7% achieved by conservative therapy [3]. Significant differences are found in the fracture morphology between complete (77.6%) and incomplete (98.2%), open (85.7%) and closed (97.1%) fractures [3]. In the case of nerve healing, the brachioradialis muscle is the first to recover, followed by the extensor carpi radialis longus, which extends and radially abducts the wrist. In addition, the Hoffmann–Tinel sign gradually distalises [10,11]. There is general consensus regarding the treatment recommendation for extremely displaced and open humeral shaft fractures with vascular or nerve lesions for early surgical exploration. However, in the grey area of less complex trauma and milder nerve deficits, controversial treatment concepts are recommended and different algorithms are proposed [12,13]. The questions remain: Which combination of fracture morphology (according to the AO = OTA/ASIF (Arbeitsgemeinschaft für Osteosynthesefragen/Orthopaedic Trauma Association/Association of the Study of Internal Fixation) classification) and nerve lesion (is the nerve torn?—interposed, constricted, distracted or compressed?) should be surgically explored at an early stage and, if so, which fixation method (plate, screws, cerclage, external fixator, or nail) should be chosen? With this retrospective case study, we investigate the relationship between fracture morphology and nerve findings in our own collective and show suitable treatment methods to contribute to decision-making in everyday surgical practice.

## 2. Materials and Methods

This case study has been approved by the institutional ethical committee. It is a retrospective review of patients with humeral shaft fracture and accompanying radial nerve lesions from Ulm University Medical Center from 2018 to 2022. The number of cases was determined by our statistics department for a period of five years. The data collection was based on our hospital information system (SAP IS-H, Germany). The case search was performed with the following ICD-10 codes:

➢ S42.3—Fracture of humeral shaft incl. humerus o.n.a., multiple shaft fractures, humerus o.n.a.
➢ S42.40—Part unspecified, incl. distal end o.n.a.
➢ S42.41—Supracondylar.
➢ S44.2—Injury of the radial nerve at the level of the upper arm.

Inclusion criteria were all acute traumatic cases of simultaneous humeral shaft fracture and symptomatic nerve lesion according to the above-mentioned ICD 10 codes from a patient age of 18 years. Exclusion criteria were patients under the age of 18, polytraumatised patients, open and pathological fractures, amputation injuries, unassessable central or peripheral neurology and underlying neurological diseases, pre-existing extremity injuries and diseases as well as functional impairment of the arm in question, known lesions of the radial nerve or brachial plexus, local infections and tumour manifestations as well as incompliance.

A descriptive data collection with statistical formation of mean values and percentages as well as tabular and graphical representation with comparative distribution was carried out. Demographic characteristics such as patient age and gender were analysed. The clinical symptoms of the radial nerve lesion, such as hypaesthesia and palsy, were recorded. Humeral shaft fractures were categorised according to the AO classification. In addition, the intraoperative macroscopic nerve findings (severed or injured—interposed, constricted, distracted or compressed) were found. The surgical procedures used with regard to nerve findings (neurolysis, nerve grafting or tendon transfer) and fracture characteristics (fixation by nail, plate, screw, cerclage or external fixator) were recorded. In addition, nerve healing was followed up with the time of restitutio ad integrum or last consultation with remaining deficit.

With regard to the retrospective study design, the follow-up of the cases was based on the available records. The follow-up treatment included physiotherapy, ergotherapy, positioning splints and electrotherapy. Patients were usually followed up at 2, 4, 6 and 12 weeks postoperatively. A clinical examination was performed routinely, with suture traction after 2 weeks and with X-ray control after 6 and 12 weeks. In addition, measurements of nerve conduction velocity and electromyography were repeated within these periods. Further follow-up controls were carried out if complaints persisted or if there was a residual functional deficit.

**Figure 1:**
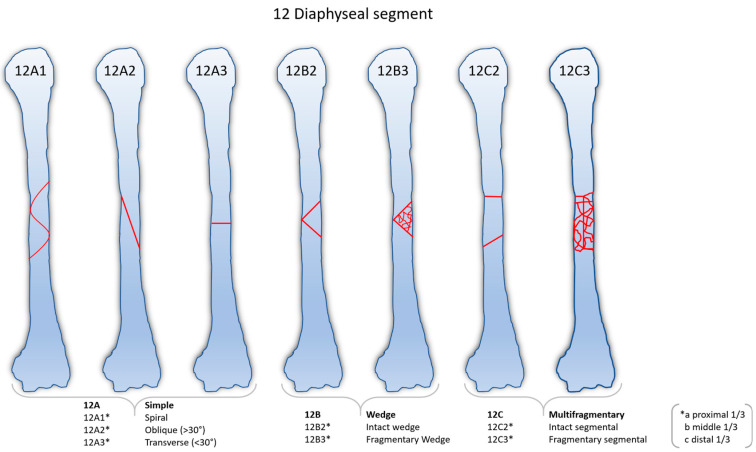
Classification of humeral shaft fractures (12 diaphyseal segments) according to the AO.

Fracture classification was based on preoperative radiographs and computed tomography scans according to the AO as shown in Figure 1.

To illustrate some cases, the Section 3 shows composite figures of preoperative X-ray, CT examination, intraoperatively photographed nerve findings and fracture osteosynthesis as well as postoperative X-ray control.

## 3. Results

From January 2018 to December 2022, a total of 804 patients (463 women and 341 men) with humerus shaft fractures were treated at our hospital. A symptomatic lesion of the radial nerve was found in 33 patients (4.1%). In 17 of the 33 patients (2.1% of the 804 patients), the clinical symptoms of the nerve lesion were found to be clearly primary, existing since the accident.

**Figure 2:**
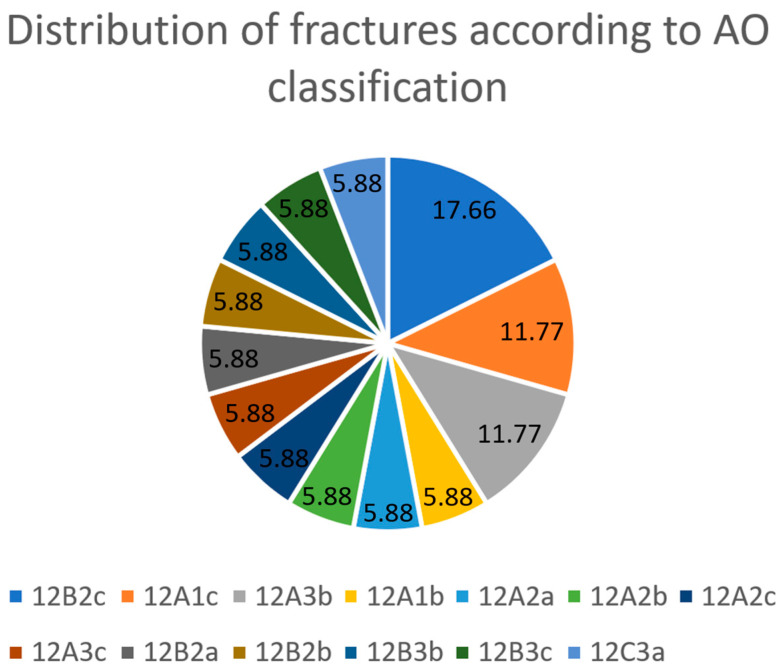
The diagram illustrates the broad and inhomogeneous distribution of AO fracture types in the 17 patients in percentages.

AO classification showed three 12B2c fractures, two 12A1c fractures, two 12A3b fractures and one each of 12A1b, 12A2a, 12A2b, 12A2c, 12A3c, 12B2a, 12B2b, 12B3b, 12B3c and 12C3a fractures. The distribution is shown in Figure 2.

Preoperatively, seventeen patients had hypesthesia and palsy. According to the operative reports, the radial nerve was found to be distracted eleven times, bony interposed three times and soft tissue constricted/compressed three times.

The radial nerve was always surgically explored and a neurolysis was performed. In three cases, the fracture was fixed with closed reduction and intramedullary nailing, and one case of open reduction and intramedullary nailing was found. Open reduction and internal fixation with a locking plate were performed in 13 cases. In addition, cerclage wiring was performed in four cases. In one case, the fracture was initially stabilised with external fixation.

There was no case of traumatic nerve transection. After surgical exploration and neurolysis, all patients underwent physiotherapy and clinical neurological follow-up.

**Figure 3:**
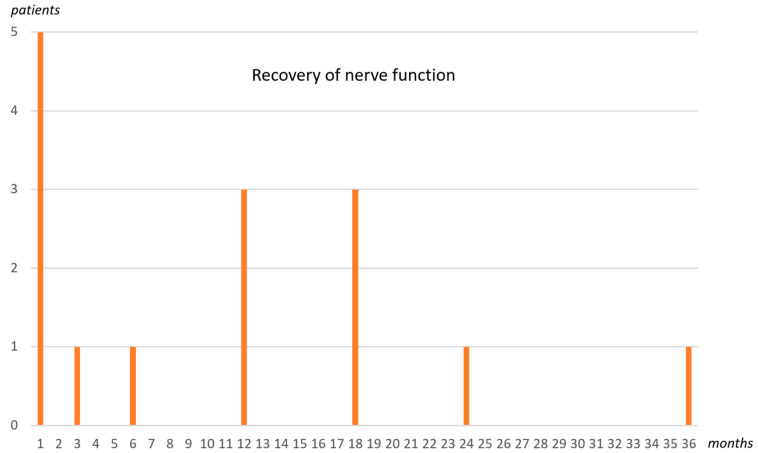
This diagram shows the recovery of nerve function over time. Three of the fifteen patients still had mild hypesthesia in the thumb area after 18 months.

Complete recovery of nerve function was seen in 12 cases within 1 to 36 months. Two patients were lost in follow-up, and three patients still showed mild hypesthesia in the thumb area after 18 months. The patient data are shown in Table 1. Figure 3 shows the recovery of nerve function in the 15 patients over time.

**Figure 4:**
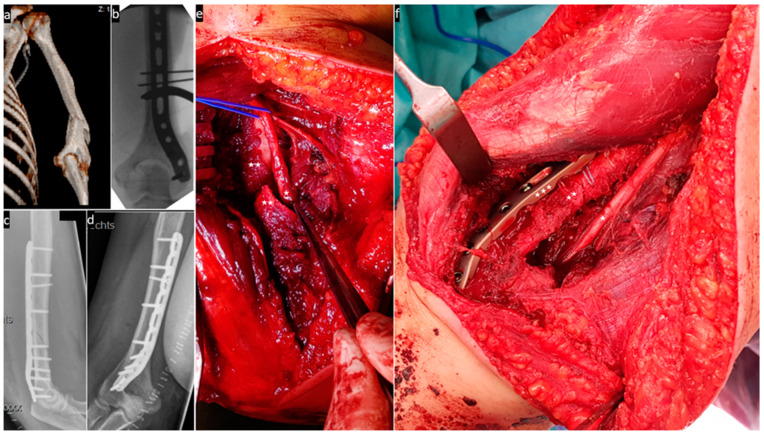
(**a**) Shows the fracture in 3D reconstruction; (**b**) intraoperative X-ray control; (**c**,**d**) postoperative X-ray control; (**e**) the radial nerve (one asterisk) is interposed between the fracture fragments (two asterisks); and (**f**) the fracture (two asterisks) is stabilised with a locking plate (three asterisks) and suture cerclages and the nerve (one asterisk) is neurolysed.

**Figure 5:**
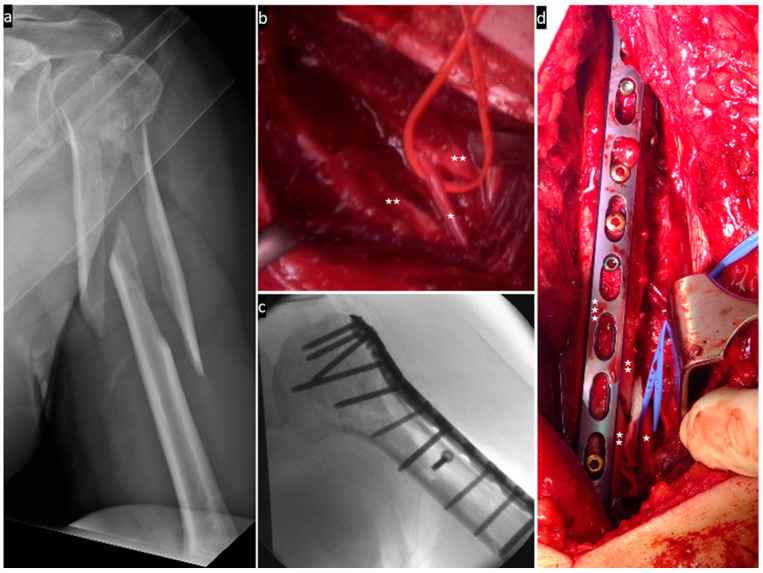
(**a**) Preoperative X-ray of the proximal fracture; (**b**) the nerve (one asterisk) is interposed between the bone fragments (two asterisks); (**c**) intraoperative X-ray control; and (**d**) the nerve (one asterisk) is now neurolysed and separated from the fracture (two asterisks) stabilised with a locking plate (three asterisks) by an absorbable mesh.

**Figure 6:**
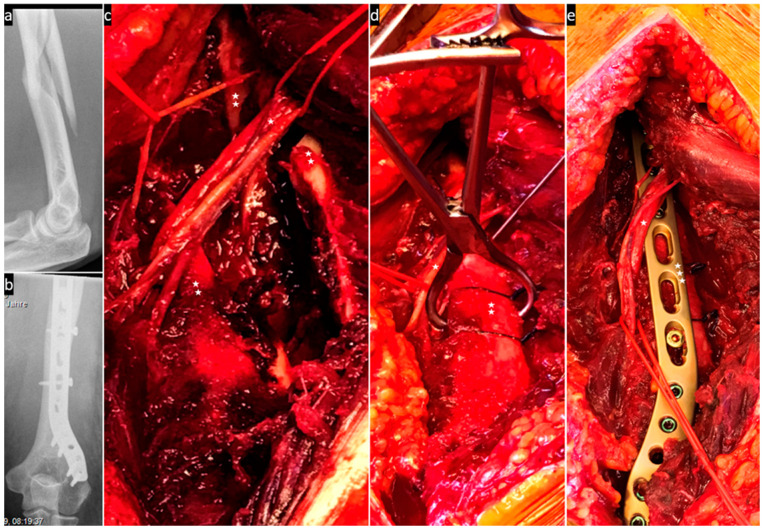
(**a**) Preoperative X-ray of the fracture; (**b**) postoperative X-ray control; (**c**) posterior approach. The interposed nerve (one asterisk) is lifted out of the multi-fragment fracture (two asterisks). (**d**) The nerve (one asterisk) lies relaxed on the repositioned fracture (two asterisks), which is temporarily secured with a clamp, a wire and two resorbable suture cerclages. (**e**) The nerve (one asterisk) crosses the locking plate (three asterisks) without tension.

**Figure 7:**
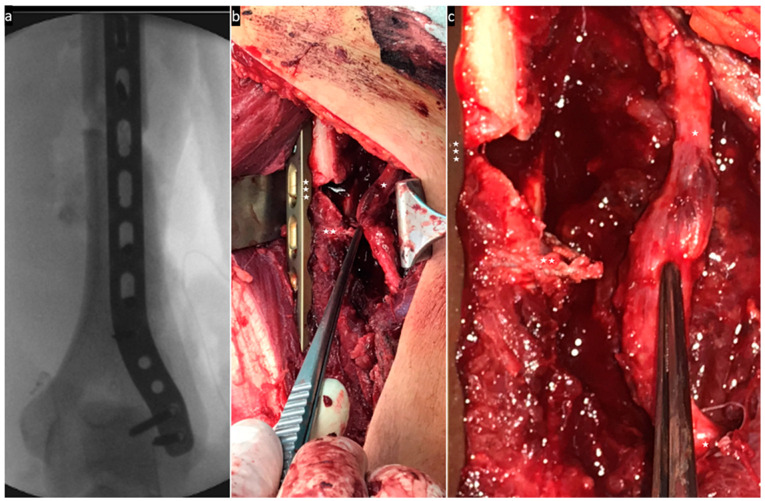
(**a**) Intraoperative X-ray control of the locking plate osteosynthesis. (**b**) The nerve (one asterisk) is clearly constricted by a periosteal cord (two asterisks) in the fracture area with plate osteosynthesis in place (three asterisks). (**c**) The connective tissue cord (two asterisks) in the fracture area next to the plate osteosynthesis (three asterisks) was severed. The neurolysed nerve (one asterisk) is intact in its continuity, but injured by the constriction.

The following images (Figure 4, Figure 5, Figure 6 and Figure 7) illustrate the intraoperative situation in radial nerve affections with distraction, interposition and constriction/compression due to axial deviation, bone fragments and connective tissue strands/hematomas.

## 4. Discussion

The incidence of primary radial palsy in humeral shaft fractures is about 11.8% (but varies widely from 2 to 17%) [2,3,7]. The literature recommends various surgical and conservative treatment methods, but one searches in vain for a guideline [12]. From January 2018 to December 2022, a total of 804 patients (463 women and 341 men) with humerus shaft fractures were treated at our hospital. Symptomatic lesions of the radial nerve were found in 33 patients (4.1%). A definite primary lesion with documented radial nerve palsy directly following the accident was found in only 17 patients (2.1%), which is less than reported in the literature. In the remaining 16 patients of our collective, the nerve lesion was secondary, detected/documented in the course of treatment. Hendrickx et al. report a secondary radial nerve palsy of 3% in their study, which is in line with our findings [7].

The distribution of primary to secondary radial palsies is reported in the literature to be 80% to 20% [14]. However, identifying and specifying clinical symptoms can sometimes be challenging. Initial examination may be obscured by pain, shock and fear; on the other hand, severe accompanying injuries may make selective detection of nerve damage difficult. The human factor is another variable for the identification of nerve damage in both the patient and the physician. In addition, nerve damage may be worsened by instability and hematoma in the fracture area. Evidence of this is lacking in the current literature.

According to the literature, radial palsy occurs most frequently in humerus fractures in the middle of the shaft and the transition to the distal third [3]. In addition, the risk is significantly increased for spiral and transverse fractures compared to oblique and comminuted fractures [3]. In our study, fracture classification by AO revealed three 12B2c fractures, two 12A1c fractures, two 12A3b fractures and one each of 12A1b, 12A2a, 12A2b, 12A2c, 12A3c, 12B2a, 12B2b, 12B3b, 12B3c and 12C3a fractures. We hereby see a broad, inhomogeneous distribution. Although the current literature no longer reports a significantly increased incidence of radial palsy in Holstein–Lewis fractures (12A1c), this fracture type is among the second most common in our small collective [3,15].

Primary radial palsies can be caused by distraction, interposition, constriction/compression and transection of the radial nerve [7]. Bony nerve interposition is reported with an incidence of 6–25% and nerve laceration with an incidence of 20–40% [3]. In 2020, Hegemann et al. reviewed 200 cases of nerve palsy in humeral shaft fractures with an intraoperative finding of 35% with no obvious lesions, 30% with neurapraxia and structural damage in 35% [16]. According to the operative reports in our collective, the radial nerve was found to be distracted eleven times, bony interposed three times and soft tissue constricted/compressed three times. This confirmed the indication for early exploration and neurolysis. Open reduction and locking plate osteosynthesis were chosen as the most common surgical procedures because the surgical approach was already given by nerve exploration and neurolysis. In addition, no complete traumatic nerve transection was found in any case.

In the literature, the spontaneous recovery rate is described as high. While a 98% recovery rate is indicated for frequent low-energy traumas, 71% is reported for high-energy trauma and open fractures [3,17]. The recovery rate of the radial nerve after surgical neurolysis and subsequent physiotherapy was also high in our study. Complete recovery of nerve function was seen from 1 to 36 months in 12 cases. Two patients could not be followed up and three patients still showed mild hypesthesia in the thumb area after 18 months. The overall convalescence time was very variable and, in some cases, quite long.

When an indication for osteosynthesis exists with primary nerve palsy, open reduction and plate fixation with nerve exposure are recommended by some authors [7,16]. Indications for humeral shaft fracture fixation include open and pathological fractures, massive soft tissue damage or bone defects, penetrating injuries and gunshot wounds, and significant deformities with shortening at ad latus displacement and axial deviations greater than 20°. In addition, osteosynthesis is recommended for polytrauma, bilateral fractures, vascular injuries and floating elbows [18]. Other indications for early nerve exploration include avoiding further nerve damage (laceration, entrapment) by stabilising the mobile fracture ends and minimising excessive fibrosis [19].

If there is no indication for immediate surgical fracture stabilisation in an acute traumatic humerus fracture with primary radial nerve palsy, such as in closed, nondisplaced fractures without significant axial or rotational deviation, there are both the options of early surgical nerve exploration and expectant observation. In favour of early exploration is the above-mentioned possibility of preventing secondary nerve damage as well as the precise assessment of the existing nerve lesion with direct possible repair, which in such a case leads to a significantly better outcome (90% recovery < 3 weeks vs. 68% recovery > 8 weeks) [19]. The high rate of spontaneous recovery (whether with or without neurolysis with rates from 73 to 92%) and the avoidance of surgical complications including secondary nerve damage such as neurapraxia or injury to the accompanying vessels/neurilemma sheath are arguments for a wait-and-see strategy [7]. In addition, ultrasound can be used to gently examine and evaluate the radial nerve with a sensitivity of up to 89% and a specificity of up to 95% [20,21]. For clinical assessment of nerve recovery, Tinel’s sign can be performed every 4 weeks [10] and an EMG examination of the brachioradialis can be performed at 3, 12 and 24 weeks [3,8,22,23]. If signs of nerve recovery remain absent until 6 months after trauma, late exploration with neurolysis, direct neurography, nerve graft, nerve transfer or tendon transfer may be indicated [13,23]. Care should be taken to ensure a tension-free situation in all circumstances.

Furthermore, there is broad consensus on the indication for early nerve exploration in secondary nerve palsies in which the radial nerve was not visualised during the initial surgery.

Due to the retrospective design and the relatively small number of cases in this study, there are of course limitations to the generalisability of the results.

## 5. Conclusions

With this study, we support the strategy of early nerve exploration and plate osteosynthesis in humeral fractures with primary radial nerve palsy when there is a clear indication for surgical fracture stabilisation. In addition, early exploration appears sensible in the case of palsies in open fractures and secondary palsy following surgery without nerve exposure as well as in the case of diagnostically recognisable nerve damage. Late nerve exploration is recommended if there are no definite signs of recovery after 6 months. An initial wait-and-see strategy with clinical observation seems reasonable for primary radial nerve palsies without indication for surgical fracture stabilisation.

## Figures and Tables

**Table 1:** The results of the 17 patients with primary radial palsy are shown. Age, sex and clinical symptoms are listed, as well as the exact fracture classification according to AO, the respective intraoperative findings of the affected nerve, surgical treatment, and time to recovery of nerve function. Two patients could not be followed up (LOFU = loss of follow-up). In three patients, mild hypesthesia in the thumb area remained until 18 months after trauma.

Patient Sex and Age (F = Female, M = Male)	Initial Radial Nerve Symptoms	AO Fracture Classification	Radial Nerve Findings Intraoperative	Fracture Treatment Method	Nerve Recovery (Restitutio Ad Integrum) in Months
F, 56	Hypesthesia + palsy	12C3a	Interposed	Neurolysis, cerclage, nail	12
M, 37	Hypesthesia + palsy	12B3b	Constricted	1. Neurolysis + fixator 2. Nail	24
F, 72	Hypesthesia + palsy	12A1b	Distracted	Neurolysis, plate	6
M, 66	Hypesthesia + palsy	12B2a	Distracted	Neurolysis, nail	1
F, 84	Hypesthesia + palsy	12A2c	Distracted	Neurolysis, plate	LOFU
F, 42	Hypesthesia + palsy	12B2c	Distracted	Neurolysis, plate	36
F, 33	Hypesthesia + palsy	12A3c	Distracted	Neurolysis, plate	1
F, 47	Hypesthesia + palsy	12A3b	Distracted	Neurolysis, plate	LOFU
F, 79	Hypesthesia + palsy	12A1c	Interposed	Neurolysis, screw, plate	18 (res. Hyp D1)
F, 22	Hypesthesia + palsy	12B2c	Distracted	Neurolysis, cerclage, screw, plate	18 (res. Hyp D1)
F, 80	Hypesthesia + palsy	12B2b	Distracted	Nail, neurolysis	12
F, 18	Hypesthesia + palsy	12B3c	Compressed	Neurolysis, plate	1
M, 36	Hypesthesia + palsy	12A2b	Constricted	Neurolysis, plate	1
F, 72	Hypesthesia + palsy	12A1c	Interposed	Neurolysis, cerclage, plate	18 (res. Hyp D1)
M, 27	Hypesthesia + palsy	12A3b	Distracted	Nail, neurolysis	3
F, 75	Hypesthesia + palsy	12A2a	Distracted	Neurolysis, cerclage, plate	12
F, 24	Hypesthesia + palsy	12B2c	Distracted	Neurolysis, plate	1

## Data Availability

All authors decided that the data and material would not be deposited in a public repository.
